# Outcomes in hospitalised patients with penicillin allergy: a systematic review and meta-analysis protocol

**DOI:** 10.1186/s13643-025-03038-0

**Published:** 2026-03-12

**Authors:** Elise Davis, Rebecca Gill, Eamonn Reda, Esther Thomas, Malikah Saba, Hanmin Chen, Tony Whitehouse

**Affiliations:** 1 University College Hospital London, London, UK; 2 Department of Critical Care, Queen Elizabeth Hospital Birmingham, University Hospitals Birmingham NHS Foundation Trust, Birmingham, UK; 3 Department of Critical Care Medicine, Ansteel Group General Hospital, Anshan, Liaoning China; 4 Institute of Inflammation and Ageing, University of Birmingham, Birmingham, UK

**Keywords:** Penicillin allergy, ICU, Antibiotic management, Mortality, Meta-analysis

## Abstract

**Introduction:**

When patients report a penicillin allergy (self-reported penicillin allergy, SRPA), clinicians alter their antibiotic management of the patient. However, fewer than 5% of SRPA have life-threatening reactions, and receiving narrower spectrum antibiotics may result in under treatment. Reports from community studies suggest that patients with SRPA are more likely to come to harm, but as SRPA implies exposure to healthcare, it is unclear whether additional harm is as a result of co-morbidities and the need to receive healthcare, rather than a harm in itself. This raises the question of how great an impact SRPA has on hospital and ICU mortality for patients admitted to hospital.

**Methods and analysis:**

A literature search will be performed using MEDLINE, Embase and CINAHL (via OVID) in October 2024. The eligible studies will be of adult hospitalised patients with penicillin allergy compared with their non-allergic counterparts. We will extract demographic and outcome data including whether the patients were treated in ICU and mortality (28-days, in-ICU or in-hospital).

If possible, the results will be pooled for a meta-analysis by combining the all-cause mortality of patients at 28 days, in-ICU or in-hospital. Heterogeneity will be assessed using the *I*^2^ or *H*^2^ statistics if the number of included studies is less than 10.

**Discussion:**

Previous reports of poor outcomes from SRPA are often based around increased risk of increased antibiotic use and antibiotic resistance. The requirement of SRPA to develop where there is exposure to healthcare and co-morbidities may mean that these outcomes are derived from poorer health rather than from SRPA perse. The impact of SRPA on hospitalised patients is less understood, with outcomes that are well-defined, such as mortality. This review will outline the differences that exist, and a meta-analysis may define the size of any differences.

**Systematic review registration:**

PROSPERO CRD42024537658.

**Supplementary Information:**

The online version contains supplementary material available at 10.1186/s13643-025-03038-0.

## Background

Penicillins are the most commonly cited drug allergy [1]. Between 8 and 25% of patients in hospital declare a penicillin allergy [2], although the generally accepted rate is probably 5% [3]. Few patients who report a penicillin allergy (self-reported penicillin allergy, SRPA) have a true IgE-mediated allergy, but many report penicillin allergy because they experience gastrointestinal upset or cutaneous rashes, which are often not immune mediated and due to other causes [4]. Whatever the manifestation of SRPA, clinicians frequently use alternative antibiotics or are reluctant to prescribe, leaving patients exposed to a greater risk from severe infection.

Studies have suggested that patients with SRPA receive second-line, broad-spectrum antibiotics and are at higher risk of infections from antimicrobial resistance [2, 4]. Furthermore, SRPA has been associated with a higher risk of therapeutic failure, longer in-hospital length of stay, increased risk of readmission [5] and increased costs [6]. Concerns that patients with SRPA have poorer outcomes have not always been reflected in hospitalised patients and in studies using outcomes such as mortality. A recent longer-term study following 20,092 adult patients for up to 12 years [7] reported that beta-lactam allergies were not associated with increased mortality (OR: 1.02, 95% CI: 0.96–1.09).

It is assumed that studies reporting outcomes from community SRPA can be applied to hospitalised patients and vice versa. SRPA, by definition, only arises because of contact with healthcare and is more likely with many chronic conditions [8, 9]. Many of the reported outcomes for community studies (increased hospital admission, increased risk of antimicrobial resistance or increased use of antibiotics) may reflect those additional comorbidities rather than poorer outcomes as a result of alternative antibiotic use. Equally, increased antibiotic use may come about because of clinician anxiety rather than treatment failure. Studies with well-defined outcomes, such as mortality, are less prominent but may not follow the findings of more subjective outcomes.

This systematic review aims to congregate the data that has been published to determine if mortality (in-hospital, 28 or 90 days) in hospitalised and ICU patients admitted for any reason is greater in patients with SRPA compared with those without. Where possible, other clinically relevant data, including length of stay and cure rates, will be extracted. This review will aim to report the mortality risk associated with self-reported penicillin allergy and, where possible, clarify the size of the effects of SRPA compared with non-SRPA.

## Methods

This systematic review is registered on the International Prospective Register of Systematic Reviews (PROSPERO) (ID: CRD42024537658) [10], and this protocol is presented in line with PRISMA-P (Preferred Reporting Items for Systematic Reviews and Meta-Analyses Protocols) guidance. The resulting review will be published in line with PRISMA (Preferred Reporting Items for Systematic Reviews and Meta-Analyses) reporting guidance [11].

### Eligibility criteria

- Population: patients admitted to hospital for any reason.
- Exposure: adult (age > 18 years) Patients with SRPA.
- Comparator: patients without SRPA.
- Outcomes: one or more of the following outcomes:
  - ◦ Primary outcome: mortality expressed as in hospital, in ICU, 28-day or 90-day.
  - ◦ Secondary outcomes: length of hospital stay and re-infection rates.
- Study type: cohort and case-controlled studies. Relevant systematic reviews will be included only for the purpose of identifying primary studies not found through the searches.

### Information sources

A literature search will be performed using MEDLINE, Embase and CINAHL (via OVID) with no language or publication status restrictions to identify articles published up until September 2024. Relevant systematic reviews will be identified in Epistemonikos.

The ethos of the strategy will combine terms for penicillins AND Allergies/Hypersensitivity AND a range of terms covering the relevant outcomes.

Where relevant, both free text and index terms (MeSH/Emtree) will be used in search strategies, and the search terms will be adapted to each database.

The strategy to be applied to MEDLINE is in supplementary material.

### Study records

Search results will be imported into Endnote (Clarivate), duplicates removed automatically, and then uploaded to Rayyan [12] to facilitate study selection. Each title and abstract will be screened for relevance against the eligibility criteria by two reviewers independently from the panel of reviewers (ED, RG, ER, ET, MS). Full text copies of relevant articles will be obtained and assessed against the full eligibility criteria for inclusion in the review by two reviewers independently. Disagreement in decisions between reviewers will be resolved through discussion, and if required, with a third reviewer (TW). Reasons for exclusion at the full text stage will be noted. The selection process will be graphically illustrated using a PRISMA flow diagram [13].

The screening and selection process will be piloted to test applicability and uniformity between reviewers.

#### Data extraction

Data will be extracted from included studies by one reviewer and checked by a second from the group using an adapted (to type of study design) Cochrane Public Health Group data extraction form. The extracted data will be checked by an independent reviewer (HC). Extracted data will include, but not be limited to:

Study characteristics:

- Article title, author, and year
- Country and setting
- Study population characteristics (sex, age, co-morbidities)
- Inclusion and exclusion criteria
- Funding sources

Screening details:

- Definition of penicillin allergy
- Screening of cases
- Data sources

#### Health outcomes

Primary outcome:

- Mortality (in-hospital, ICU, 28-day, 90-day)

Secondary outcomes:

- Length of hospital stay
- Antibiotic usage
- Incidence of resistant bacterial infections
- Readmission rates

Given the likely variability between studies included in the review, in terms of design, population, intervention, comparator, outcomes and data type, the data extraction process will be piloted and then modified using examples from the range of differing studies included.

#### Quality and bias assessment

Studies will be assessed for risk of bias by reviewers and graded using the ROBINS-I tool [14]. Allergy status will be considered as ‘exposure’ and will be used to compare outcomes in two different populations: those with self-declared penicillin allergy and those with undeclared penicillin allergy. Bias will be assessed independently by two reviewers. It is unlikely that there will be any randomised trials, but if any are extracted, the Cochrane Collaboration’s Risk of Bias Tool (2nd version) [15] will be used.

#### Data synthesis

Data will be reported in a mixture of formats for the same outcome (e.g. continuous data mean, median, risks/relative risks, odds ratios). Attempts will be made to meta-analyse through aggregation of data at a study level.

#### Data collection and tabulation

Data from each study will be organised to capture the following key characteristics:

- Study design: categorise studies as cohort studies or case–control studies.
- Population: record details such as age, gender, and comorbidities.
- Penicillin allergy status: note the number of patients with and without penicillin allergy.
- Outcome measures: include metrics such as mortality rate, length of hospital stay, antibiotic usage, emergence of resistant bacteria, and readmission rates. We will also extract rates of infection recurrence if reported.

#### Data grouping and analysis

- Study design: matched studies and cohort studies.
- Population characteristics: stratified data based on age groups, gender, base speciality and presence of comorbidities. Country of study
- Outcome measurement time frames: categorise studies into short-term (e.g. within 30 days) and long-term (e.g. beyond 30 days) outcomes.

#### Outcome measures

To facilitate aggregation and meta-analysis, we will calculate consistent statistics across all studies. Where percentages are reported, we will calculate index values by multiplying percentages by the number of participants in the group. For continuous data, we will calculate the mean difference or standardised mean difference. For categorical data, we will calculate odds ratios.

Clinical and methodological homogeneity will be assessed so that the feasibility for meta-analysis, where two or more studies report data in the same format, can be undertaken. Any meta-analysis will be undertaken using a random effects model. Determination of the level of between-study variation not attributable to chance will be calculated and displayed as an *I*^2^ value with a 95% confidence interval.

The potential for sensitivity analysis or the assessment for the existence of small study effects using a funnel plot may be feasible. Risk of bias information will be used descriptively to assess possible biases and understand the significances for each outcome, whether a meta-analysis is undertaken or not. Recommendation for the improved conduct of studies in the field will be made.

It is unlikely that there will be interventional trials in our search but if necessary, GRADE methodology [16] will be used to evaluate the certainty (or confidence) in the body of evidence for each outcome (e.g. high, moderate, low, or very low confidence). An explanation of reasons for rating down (or rating up) the certainty of evidence (such as in footnotes to an evidence summary table) will be provided.

## Discussion

The presence of penicillin allergy changes prescribing practise and the behaviour of clinicians. However, the presence of penicillin allergy must occur because of exposure to antibiotics and medical services and, when compared to a non-SRPA group, the difference in outcome may represent differences in co-morbidities, exposure to healthcare and exposure to antibiotics. The amount of these exposures is frequently not recorded and included in retrospective studies. This provides a complex impact on outcomes since, whilst SRPA necessitates exposure to healthcare, exposure may lead to better control of comorbidities (which is unmeasured and unadjusted in a study) or more diagnosed comorbidities compared with the non-SRPA population (which may be a measure co-variable). Previous community studies of SRPA have suggested poorer outcomes from SRPA but may not have been able to account for the variables that have caused SRPA to come about. The risks of unmeasured biases built into reporting outcomes from SRPA may be reduced in the ICU as admission is selected based on the ability of the ICU to improve the patient’s condition. Furthermore, it is possible that there will be differences between national healthcare systems and access to healthcare as ICU admission decisions differ between nations.

The declaration of a penicillin allergy alters the way that healthcare professionals interact with the patient. However, the protocolisation of care that is present in ICU combined with the common reporting of outcomes that are easily measurable (mortality) makes the ICU a unique environment to study SRPA. Withdrawal of life-supporting care in ICU and decisions around referral or admission to ICU are made on the patient’s condition and not their SRPA status, reducing certain biases. There will, however, be major differences in the antibiotic management of patients which may explain any differences in mortality. Leibovici [17] has suggested that penicillin allergy may reflect a “healthy user” bias where the flagging of an allergy brings special attention to the patient in contrast to their peers in the non-SRPA group. In critically ill patients in ICU, SRPA is unlikely to bring special attention to patients and may allow SRPA to be regarded as a natural experiment between different antibiotic regimens.

By systematically evaluating the existing literature, highlighting differences between healthcare systems and identifying gaps in understanding, this review may inform clinical practice, especially around antibiotic use, and guide future research. It will define the impact that SRPA presents to patients and contribute to the development of more effective strategies for managing patients with reported penicillin allergies.

## Supplementary Information

Supplementary Material 1. Search Strategy.

Supplementary Material 2. PRISMA checklist.

## Data Availability

No data has yet been generated.
