# Supplementary material for: Outcomes in hospitalised patients with penicillin allergy: a systematic review and meta-analysis protocol
Source: Syst Rev. 2026 Mar 12;15:78. doi: 10.1186/s13643-025-03038-0 (PMC12983788; doi:10.1186/s13643-025-03038-0)
Supplement: Supplementary file 1 — Supplementary Material 1. Search Strategy. [file 13643_2025_3038_MOESM1_ESM.docx]

# Supplementary Material

## Search Strategy

OVID Search:

1. penicillin*.ab, hw, sh, ti.

2. lactam*.ab, hw, sh, ti.

3. 1 or 2

4. allerg*.ab, hw, sh, ti.

5. hypersens*.ab, hw, sh, ti.

6. 4 or 5

7. mortalit*.ab, hw, sh, ti.

8. surviv*.ab, hw, sh, ti.

9. outcome*.ab, hw, sh, ti.

10. 7 or 8 or 9

11. communi*.ab, hw, sh, ti.

12. primary care.ab, hw, sh, ti.

13. 11 or 12

14. child*.ab, hw, sh, ti.

15. exp Child/

16. exp Children/

17. infan*.ab, hw, sh, ti.

18. exp Infants/

19. neonat*.ab, hw, sh, ti.

20. exp Neonates/

21. 14 or 15 or 16 or 17 or 18 or 19 or 20

22. (3 and 6 and 10) not (13 or 21)

The reference lists of included studies, and studies included in any relevant systematic reviews, will be checked for additional relevant studies.
